# Peptide immunoarrays for rationale development of vaccines with enhanced cross-reactivity

**DOI:** 10.1371/journal.pone.0330741

**Published:** 2025-09-04

**Authors:** Zoë Parker Cates, Antonio Facciuolo, Erin Scruten, Anthony Kusalik, Scott Napper

**Affiliations:** 1 Department of Computer Science, University of Saskatchewan, Saskatoon, Saskatchewan, Canada; 2 Vaccine and Infectious Disease Organization (VIDO), University of Saskatchewan, Saskatoon, Saskatchewan, Canada; 3 Department of Veterinary Microbiology, University of Saskatchewan, Saskatoon, Saskatchewan, Canada; 4 Department of Biochemistry, Microbiology and Immunology, University of Saskatchewan, Saskatoon, Saskatchewan, Canada; University of Arkansas for Medical Sciences, UNITED STATES OF AMERICA

## Abstract

Vaccines of enhanced range of protection could help to control newly emerging infectious diseases while providing greater resilience to any subsequent variants. Such “universal vaccines” are an idealized, but unrealized, goal that may benefit from unbiased, high-throughput approaches that define antibody cross-reactivity to enable rational selection of cross-protective epitopes. The priority of this investigation is to establish a pipeline for the identification and preliminary characterization of epitopes with enhanced cross-reactivity. A peptide immunoarray representing the proteomes of SARS-CoV, SARS-CoV-II, and MERS-CoV was applied to characterize spike glycoprotein-specific antibody populations within convalescence serum of SARS-CoV-II infected ferrets. Through two alternate bioinformatic approaches, twenty candidate epitopes were identified and translated into vaccines. Epitopes inducing antibodies with cross-reactivity across naturally occurring versions of spike glycoprotein, including SARS-CoV-II Delta and Omicron variants, as well as antigenically distant SARS-CoV and MERS-CoV, were identified. Working from the assumption that cross-reactivity is prerequisite for cross-protection, this highlights the opportunity and mechanisms by which immunoarrays, coupled with *in vitro* screening assays, can enable rational selection of epitopes with enhanced potential for cross-protection.

## Introduction

The emergence of severe acute respiratory syndrome coronavirus 2 (SARS-CoV-II) was responsible for an estimated 7 million deaths, an outcome which would have been far worse if not for the rapid development of safe and effective vaccines [1,2]. While these vaccines were produced with unprecedented efficiency, the period between pathogen emergence and vaccine availability highlighted a critical window of vulnerability which is inherent to reactive approaches to emerging infectious disease threats. Shortening, or ideally, eliminating, this window would mitigate the consequences of future infectious disease threats. To this goal, vaccines with a range of protection against a pathogenic family, rather than specific pathogen, could be generated and stockpiled for use at the earliest indications of an outbreak. Such an approach could ideally prevent epidemics from becoming pandemics. Vaccines with an enhanced range of protection could also provide greater resilience to emerging variants. For example, the value of the first-generation COVID-19 vaccines was soon threatened by the emergence of variants of SARS-CoV-II.

The COVID-19 vaccines differed primarily in their delivery platforms (mRNA for Pfizer and Moderna: viral vectored for Johnson &Johnson and AstraZeneca) while utilizing a common antigen; spike glycoprotein from early variants of SARS-CoV-II [3]. Spike is the protective antigen of SARS-CoV-II as well as other coronaviruses; detailed information is available on the structure-function relationship of this protein across these viruses and variants, including how escape neutralization to vaccine-induced antibodies largely reflects changes within sequences of spike [4,5]. While the first-generation vaccines [6], as well as infection with the ancestral strain [7–9], afforded protection to the early emerging variants, their effectiveness and duration of protection was compromised against later variants [10–12]. Vaccine-resistant variants diminish the value of past and future vaccine administrations. This prompted the development of second generation, bivalent vaccines that incorporated spike glycoprotein sequences that reflect the priority circulating variants [13]. While logical, this again represents a reactive approach in which vaccine development/optimization only commences once the new/evolving threat has been actualized.

A more proactive approach is to prioritize vaccines whose protection has greater independence from sequence deviations within the protective antigen. This could bypass the timeframes associated with development of novel vaccines, provide greater resilience against emerging variants, and provide a mechanism to deal with antigenically variable pathogens (AVPs), which are problematic for traditional, high-specificity vaccines [14]. While enhanced cross-reactivity may compromise the extent of protection bestowed to the individual, this could still represent an important tool to minimize the consequences of a new pathogen while providing the time to develop traditional, high-specificity vaccines. Despite their appeal, “universal vaccines” remain an unachieved goal. For example, despite efforts to develop a universal flu vaccine, novel antigens are required each year [15,16]. Currently most efforts to develop cross-reactive vaccines either target highly conserved regions or regions critical to virus function [17]. While rational strategies, it is also possible that the necessary epitopes are not biologically intuitive and may therefore benefit from unbiassed, high-throughput approaches to provide deeper characterization of the reactivities, and cross-reactivities, of antibody populations induced by infection and/or vaccination.

Peptide immunoarrays are an emerging technology for high-throughput characterization of antibody reactivities to potential antigenic sequence determinants. Immunoarrays consist of hundreds to thousands of unique peptides, typically 9–20 amino acids in length, localized to specific coordinates on a glass slide. Immunoarrays have been applied to map antigenic determinants (epitopes) of microbial proteins [18,19], provide immunosignatures of responses to vaccines and microbial pathogens [20–22] and as diagnostic tools to determine exposure to a pathogen [23]. Immunoarrays can also assist in vaccine development through evaluation of the ability of candidate vaccines to induce antibodies with reactivity to neutralizing epitopes. Finally, characterization of reactivities through immunoarrays have shaped understandings of antibody-antigen interactions [24].

Our priority is to establish a pipeline for the rapid development and characterization of candidate vaccines with an expanded range of cross-reactivity to a known protective antigen. This investigation utilizes a commercial peptide immunoarray representing the proteomes of several coronaviruses, including Severe Acute Respiratory Syndrome coronavirus (SARS-CoV-I), SARS-CoV-II, and Middle East Respiratory Syndrome coronavirus (MERS-CoV), as overlapping peptides to facilitate mapping of reactivities to specific epitopes [23]. The array was applied to characterize serum antibody populations of SARS-CoV-II convalescent ferrets to identify cross-reactive epitopes within spike glycoprotein. Ferrets are a well-established animal model for SARS-CoV-II infection [25] and spike glycoprotein is the protective antigen of commercialized COVID vaccines [26].

**Fig 1:**
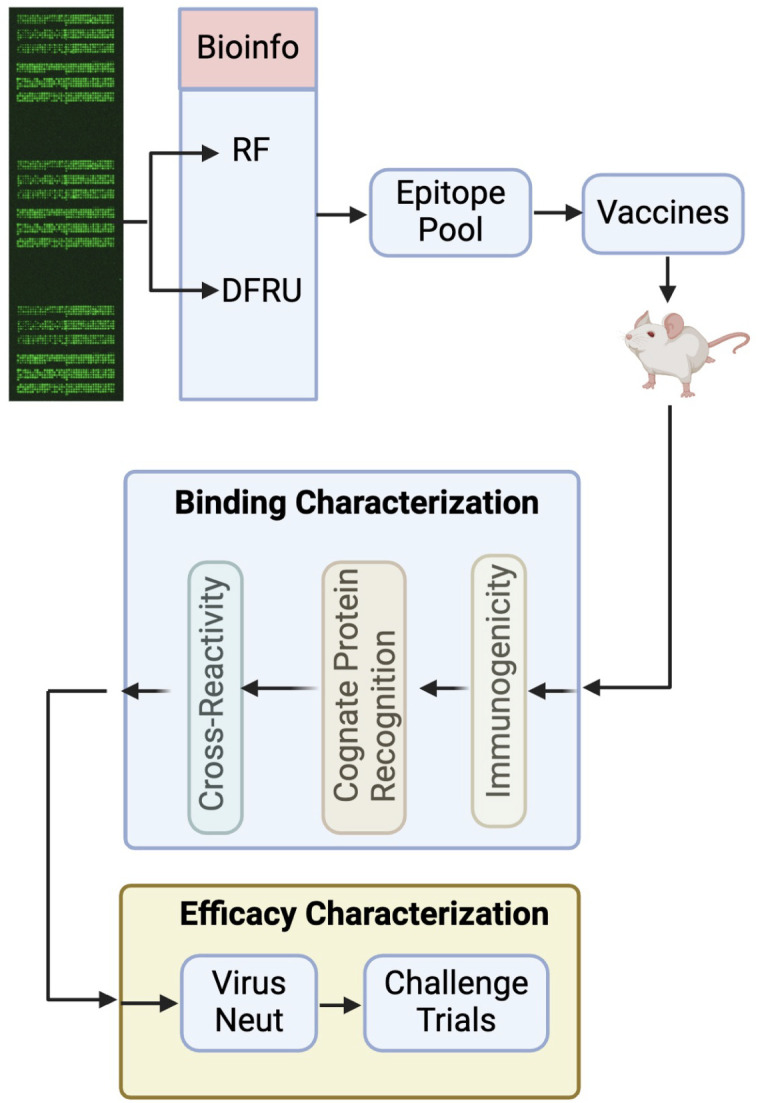
Pipeline for Application of Immunoarrays to Develop Cross-Reactive Vaccines.

Two bioinformatic approaches were applied to the immunoarray data. Delta Relative Fluorescence Units identifies peptides with greatest differential reactivity between treatment and control, as well as high relative degree of reactivity and sequence conservation among epitopes. Random Forest utilizes machine learning to identify peptides that best distinguish pre- and post-infection samples. From these approaches, a panel of twenty epitopes was identified and translated into vaccines which were administered to mice. Vaccine-induced antibodies were interrogated through *in vitro* assays to quantify cross-reactivities with naturally occurring versions of spike. A schematic overview of the pipeline is presented (Fig 1). Epitopes were identified which achieved cross-reactivity with SARS-CoV-II Delta and Omicron variants, as well as more evolutionarily distant SARS and MERS, including those recognizing non-conserved epitopes. Protective efficiency has yet to be determined, but working from the assumption that cross-reactivity is prerequisite for cross-protection, the results highlight the opportunity, and mechanisms, by which immunoarrays, coupled with *in vitro* screening assays, can enable rational selection and subsequent streamlining of epitopes with enhanced potential for cross-protection.

## Materials and methods

### Animal use and ethics statement

All experiments were completed at the University of Saskatchewan following regulations established by the Canadian Council on Animal Care and approved by the University of Saskatchewan Animal Care Committee (Protocol #20200016). As the mice were only vaccinated, and blood was collected via tail vein, no anesthesia was required for these procedures. To alleviate any discomfort the animals may have felt during this time, all procedures were performed by qualified animal care technicians and veterinarians. If an animal exhibited any pain or distress, they were monitored and a checklist was followed to measure the pain, and, if warranted, humanely euthanized with deep isoflurane anesthesia with death confirmed by bleeding out through cardiac puncture.

### Peptide immunoarray assay

RepliTope™ Antigen Collection Pan-Coronavirus (Product Code: RT-HD-CoV2) microarrays were purchased from JPT Peptide Technologies (Berlin, Germany). Each array consists of 4416 unique peptides covering the full proteome of ancestral Wuhan SARS-CoV-II (accession YP_009724390.1), and spike glycoprotein, nucleoprotein, envelope small membrane protein, and membrane protein of SARS-CoV (accession NP_828851.1), MERS-CoV (accession AFS88936.1), and common cold coronaviruses HCoV-229E (accession P15423.1) and HCoV-OC43 (accession P36334.1). Spike glycoprotein accession numbers for SARS-CoV-II (YP_009724390.1), SARS-CoV (YP_009825051.), MERS-CoV (AFS88936.1), HCoV-229E (P15423.1), and HCoV-OC43 (P36334.1) are provided. Each protein target is represented by consecutive 15-mer peptides with 11 amino acid overlap and printed in triplicate. All incubation steps were performed at room temperature on a rotating shaker. Peptide microarrays were blocked in Tris-buffered saline (TBS), pH 7.2 supplemented with 0.05% v/v Tween-20 (TBS-T) and 3% w/v bovine serum albumin fraction V (BSA; diluent) for 30 min. Serum was diluted 1:100 in diluent and incubated for 2 h. Each array was washed with 5 exchanges of TBS-T, and once with sterile deionized distilled water. Serum IgG antibodies were detected using Alexa Fluor 647 conjugated goat anti-ferret IgG, Fc(gamma) fragment specific antibody (Jackson ImmunoResearch, 109-605-098) diluted to 1 µg/mL in diluent and incubated for 45 min in the dark. Washes were carried out as previously described, and slides dried by centrifugation for 5 min at 800 × g.

### Immunoarray image acquisition

Peptide arrays were imaged using a GenePix Professional 4200A microarray scanner (MDS Analytical Technologies, Toronto, ON, Canada) equipped with a 635 nm laser and fluorescence captured using a 655–695 nm filter. Images were scanned at 10 μm resolution and data acquired using GenePix software (version 7.0). A representative example of an array image is included in the supplementary data (S1 Fig). Peptide microarray data is available in the publicly accessible repository figshare.com using the DOI 10.6084/m9.figshare.29275199. Raw data was processed with the EPIphany software platform [27] using default parameters to yield a table of intensities referred to below as the ferret dataset. In the table, each row is an individual peptide, and columns consist of the signal intensity (mean over 3 replicates) resulting from antibody binding reactivities in serum collected pre- and post-infection for each ferret. Thus, each row includes 16 signal intensities, 2 (pre- and post-infection) for each of 8 ferrets.

### Peptide epitope selection

From the immunoarray data, peptides were selected using two different methods termed DRFU (Delta Relative Fluorescence Units) and RF (Random Forest). The DRFU method and calculations allow identification of peptides with high differences in immunoarray reactivity in pre-immune and convalescent serum collected from ferrets challenged with ancestral Wuhan strain SARS-CoV-II. The RF method ranks the immunoarray peptides by importance when classifying samples as pre-immune or convalescent using a random-forest machine-learning model. The specific details of each approach are described below.

### Multiple sequence alignment

A multiple sequence alignment (MSA) of the spike protein sequences for SARS-CoV-I, SARS-CoV-II, MERS-CoV, HCoV-OC43, and HCoV-229E was created using default settings for the Clustal Omega program [28] available at EMBL-EBI (https://www.ebi.ac.uk/jdispatcher/msa/clustalo). After performing this alignment, HCoV-OC43, and HCoV-229E were removed from further consideration to focus on the more highly conserved SARS-CoV-I, SARS-CoV-II, and MERS-CoV sequences.

### Epitope selection through Delta Relative Fluorescence Units

DRFU (Delta Relative Fluorescence Units) refers to the change in intensity of spots on the immunoarray between control (pre-infection) and treatment (post-infection convalescent) groups, calculated using a difference of means. The DRFU calculation is demonstrated in Equation 1, where $d_{p}$ is the DRFU for a given peptide *p*, $\sum t_{p}$ is the sum of all treatment intensity values for *p*, $\sum c_{p}$ is the sum of all control intensity values for that peptide, and *n*_*c*_ and *n*_*t*_ are the number of control and treatment values, respectively.


$$d_{p}=\frac{\sum t_{p}}{n_{t}}-\;\frac{\sum c_{p}}{n_{c}}$$

Here *n*_*c*_ = *n*_*t*_ = 8.

DRFUs were calculated for the 316 peptides from the spike protein of SARS-CoV-II. Peptides with a DRFU of less than 1000 were eliminated from further analysis. Based on the MSA, and to focus on sequences that are conserved across viruses, peptides that included more than 2 gaps in the alignment between SARS-CoV-II and MERS-CoV, or between SARS-CoV-II and SARS-CoV-I, were excluded from further analysis. From the original 316 spike peptides, 66 peptides continued to the next step of analysis.

Next, the DRFUs for the segments of the SARS-CoV-I and MERS-CoV sequences that aligned with the SARS-CoV-II candidates were obtained. The MSA between the SARS-CoV-II, SARS-CoV-I and MERS-CoV sequences was used for this. If the SARS-CoV-I and MERS-CoV segments were not an exact match to peptides available on the array, then the DRFUs for the SARS-CoV-I and MERS-CoV segments were interpolated using a linear combination of the values of available array peptides that overlapped before and after the aligned segments. The weights in this linear combination were based on the degree of overlap between the probes from MERS-CoV or SARS-CoV-I and the peptide in question from SARS-CoV-II.

The percent reactivity conserved between SARS-CoV-II and SARS-CoV-I, as well as between SARS-CoV-II and MERS-CoV, were calculated for each SARS-CoV-II candidate as follows, where *v*_*drfu*_ is the DRFU of either SARS-CoV-I or MERS-CoV, *s*_*drfu*_ is the DRFU of SARS-CoV-II, and *p*_*reac*_ is the percent reactivity conserved:


$$p_{reac}=\frac{v_{drfu}}{s_{drfu}}*100$$

To determine the sequence conservation, the relative sum-of-pairs (RSOP) was calculated for each aligned SARS-CoV-I to SARS-CoV-II peptide pair and MERS-CoV to SARS-CoV-II peptide pair using the BLOSUM90 (B90) substitution matrix. The length of a pairwise alignment for a particular SARS-CoV-II peptide varied between 15 and 17 amino acids in length, as up to 2 gaps were permitted. B90 was used to calculate a score for every column in the alignment, with a gap opening penalty of −10 and a gap extension penalty of −1. These column scores were added, resulting in a sum-of-pairs. The RSOP was obtained by dividing the sum-of-pairs by the length of the alignment. Because some scores were initially between −3 and 0, + 3 was added to every RSOP to avoid future divide-by-zero errors. In this way, two RSOP values were obtained for each SARS-CoV-II peptide – one for its alignment with SARS-CoV-I, and one its alignment with MERS- CoV. Equation 2 portrays this calculation as performed for SARS-CoV-I where *n* indicates an alignment position. The same equation was used for MERS-CoV where its sequence replaces that of SARS-CoV-I.


$$RSOP=\frac{\sum_{n=1}^{length}{B90(SARS-CoV-II,\;SARS-CoV-I)}_{n}}{length}+3$$

Finally, each percent reactivity conserved value was divided by the corresponding RSOP value to determine two final scores for each SARS-CoV-II spike peptide with regards to SARS-CoV-I and MERS-CoV, respectively.

### Epitope selection through random forest

The random forest model was implemented in R, using the CRAN randomForest library (https://cran.r-project.org/web/packages/randomForest/index.html). Application of the random forest algorithm to the ferret immunoarray data allowed us to determine which peptides were most important when classifying samples as either pre-infection or post-infection with SARS-CoV-II.

The input dataset consisted of the 8 pre-immune and 8 convalescent sera samples from ferrets. These samples were randomly divided using a 70−30 split. 11 serum samples (68.75%) were used to train the RF model and 5 serum samples (31.25%) were used for testing. The training data included 4 pre-immune serum samples and 7 convalescent serum samples. The testing data included 4 pre-immune serum samples and 1 convalescent serum sample. The number of variables to randomly sample as possible predictors at each branch was set to 150. The number of trees in the forest was set to 1000, and the maximum tree depth set to 8. The features were the 316 SARS-CoV-II spike peptides.

The random forest model was applied to the ferret data. After testing the features (peptides) were ranked based on two metrics that measure variable importance: mean decrease in Gini Index and mean decrease in accuracy [29,30]. Again, SARS-CoV-II peptides with more than 2 gaps in the alignment with SARS-CoV-I or MERS-CoV were removed.

### Final candidate epitope selection

To enable a reasonable number of candidate vaccines for animal testing, the DRFU and RF candidate pools were reduced by eliminating candidates within each method with significant overlap. The step size between peptide sequence fragments on the immunoarrays was 4, meaning that neighboring peptides overlapped by 11 amino acids. Because the peptide fragments were 15 amino acids in length, two candidates in proximity overlapped by 3, 7, or 11 amino acids.

Within a set of overlapping peptides consideration of reactivity via visual examination was used to choose a best representative. Bar graphs of the SARS-CoV-II DRFU and the DRFUs of the aligned SARS-CoV-I and MERS-CoV peptides were generated for each candidate. During visual inspection of these bar graphs, candidates were eliminated based on low reactivity. In cases where a SARS-CoV-I or MERS-CoV DRFU was extrapolated, the actual DRFUs for both contributing SARS-CoV-I or MERS-CoV peptides were graphed to include as much information as possible in the decision to eliminate a SARS-CoV-II peptide.

### Generation of peptide-based vaccine candidates

Peptides represented the selected epitopes were chemically synthesized by GenScript Biotech (Piscataway, New Jersey, USA) following standard manufacturing procedures. The constructs were then conjugated to keyhole limpet hemocyanin (KLH) via maleimide-based coupling.

### Mouse immunization

Balb/c mice (n = 3/group) of 5–6 weeks of age were administered three doses on Days 0, 28, and 48. Each dose consisted of 5 µg KLH-peptide fusion formulated with 30% Emulsigen-D in a total volume of 50 µL (25 µL/thigh) delivered intramuscularly. Serum samples were collected on Day 0, 28, 48, and 76.

### ELISAs

Ancestral SARS-CoV-II, Delta, Omicron, SARS-CoV-I and MERS-CoV spike proteins were commercially sourced (SinoBiological Cat. #40589-V08B1, 40589-V08B16, 40589-V08H38, 40634-V08B and 40069-V08B), as well as the peptides (Genscript). Immulon 2HB 96-well microtiter plates (Thermo Scientific Cat. #3655) were coated by adding 100 μL/well of either the spike proteins (1ug/mL) or peptides (5ug/mL) diluted in Sodium Carbonate buffer, pH 9.6 and incubated overnight at 4°C. Plates were washed with 300uL of TBS supplemented with 0.05% v/v Tween-20 (TBS-T) four times and then wells were blocked with 5% w/v skim milk in TBS-T for 1 h at room temperature (RT). Four-fold serial dilutions of serum (starting at 1:100, in TBS-T + 1% w/v skim milk) were added to duplicate wells and incubated at 37°C for 1 h. Plates were washed as described above. For detection of IgG antibodies, alkaline phosphatase conjugated, goat anti-mouse IgG (1:10,000 in diluent; KPL Cat. #5220−0355) was added to each well and incubated for 1 h at 37°C. After washing, each well was reacted with 100 μL of p-Nitrophenyl phosphate (PNPP; Sigma Cat.# N3254) diluted in diethanolamine buffer at a concentration of 1 mg/mL. Reactions were allowed to develop until the positive controls observed an absorbance of greater than 2.0 measured at 405nm with a reference at 490 nm using a SpectraMax Plus 384™ Reader (Molecular Devices). Antibody titers were determined using the reciprocal of the highest dilution that resulted in an absorbance value greater than the mean + 3 standard deviations (SD) of the absorbance value from control samples.

## Results

### Peptide immunoarrays

Immunoarray analysis was performed on serum collected from ferrets immediately prior to, and 4 weeks after, infection with ancestral Wuhan strain SARS-CoV-II. To minimize impacts of animal variability, comparisons were performed in a paired fashion to consider changes within each animal. A range of reactivities to epitopes within proteins of the different coronaviruses was observed.

### Computational and experimental selection of candidate epitopes

As described earlier, SARS-CoV-II spike peptides that involved more than 2 gaps in the alignment with corresponding SARS-CoV-I and MERS-CoV sequences or had an initial DRFU of less than 1000 were eliminated from further consideration. From the original 316 SARSCoV-2 spike peptides represented on the peptide array, 250 peptides were filtered out by the steps above and 66 peptides continued to the next step of analysis.

Scores based on the percent reactivity conserved and relative sum-of-pairs were calculated for SARS-CoV and MERS-CoV. The 66 possible candidates were ranked based on the SARS-CoV-I final score and again based on the MERS-CoV score, resulting in two different orderings of the 66 candidates. The top 25 candidates in the SARS-CoV list have 14 peptides in common with the top 25 candidates from the MERS-CoV list. Those peptides are labelled as coming from the DRFU method (Table 1).

**Table 1:** Candidates selected by the DRFU method. Candidates are sorted based on their SARS-CoV score in the first column and by their MERS-CoV score in the second column. The third column contains candidates common to both columns, sorted according to start position. The identifier of the peptide consists of the start to stop position within the sequence of the spike protein from ancestral SARS-CoV-II.

DRFU SARS-CoV Candidates	DRFU MERS-CoV Candidates	Common Candidates
0317-0331	0621-0635	0097-0111
0549-0563	0625-0639	0101-0115
0621-0635	0441-0455	0193-0207
0097-0111	0097-0111	0341-0355
0493-0507	0633-0647	0345-0359
1009-1023	0185-0199	0441-0455
0785-0799	0345-0359	0445-0459
0789-0803	0449-0463	0449-0463
0441-0455	0445-0459	0453-0467
0757-0771	0101-0115	0549-0563
0345-0359	0553-0567	0553-0567
0777-0791	0557-0571	0557-0571
0553-0567	0341-0355	0621-0635
0369-0383	0453-0467	0777-0791
0101-0115	0233-0247	
0445-0459	0549-0563	
0193-0207	0777-0791	
0449-0463	0133-0147	
0557-0571	0193-0207	
0309-0323	0033-0047	
0341-0355	0357-0371	
0781-0795	0893-0907	
0453-0467	0533-0547	
0897-0911	0349-0363	
0025-0039	0885-0899	

Next, the random forest model was applied to the ferret dataset, building a model to predict whether a given data sample came from a ferret pre-infection or post-infection. The 316 SARS-CoV-II spike peptides were features in the model. These peptides were ranked based on two different metrics that determine a feature’s importance to the model, mean decrease in Gini Index and in accuracy. Using the 3-virus multiple sequence alignment described earlier, any SARS-CoV-II peptides with more than 2 gaps in the alignment with MERS-CoV or SARS-CoV were removed. Out of the 255 remaining peptides, the top 25 candidates by each of the two variable importance metrics (Gini Index and accuracy) were compared. Fifteen candidates common to both lists were identified and continued to the final selection step (Table 2).

**Table 2:** Candidates from the RF method. The top 25 random forest candidates ranked by mean decrease in accuracy and mean decrease in Gini Index are in the first and second columns, respectively. The third column contains candidates common to both columns, sorted according to start position. The identifier of the peptide consists of the start to stop position within the sequence of the spike protein from ancestral SARS-CoV-II.

RF Accuracy Candidates	RF Gini Index Candidates	Common Candidates
0869-0883	0873-0887	0169-0183
0745-0759	1069-1083	0493-0507
1069-1083	1173-1187	0541-0555
0409-0423	0905-0919	0665-0679
1173-1187	0869-0883	0745-0759
0905-0919	0349-0363	0825-0839
1149-1163	1149-1163	0833-0847
0665-0679	0665-0679	0869-0883
0833-0847	0833-0847	0873-0887
0793-0807	0625-0639	0885-0899
1169-1183	0049-0063	0905-0919
0541-0555	0297-0311	1069-1083
0693-0707	0389-0403	1149-1163
0469-0483	0745-0759	1173-1187
0777-0791	0885-0899	1245-1259
0873-0887	0493-0507	
1125-1139	0713-0727	
1245-1259	0825-0839	
0313-0327	0541-0555	
0169-0183	0609-0623	
0957-0971	1245-1259	
0825-0839	0281-0295	
0885-0899	0169-0183	
0493-0507	0989-1003	
0549-0563	0913-0927	

The 14 DRFU and 15 RF candidates were reduced to 8 and 12 candidates, respectively, by eliminating candidates with significant overlap within each set. To determine which overlapping candidate to eliminate bar graphs of the SARS-CoV-II DRFU and the DRFUs of the aligned SARS-CoV and MERS-CoV peptides were generated for each candidate and visually inspected. Candidates were eliminated based on low reactivity. In cases where a SARS-CoV or MERS-CoV DRFU was extrapolated, the actual DRFUs for both contributing SARS-CoV or MERS-CoV peptides were graphed to include as much information as possible in the decision to eliminate a SARS-CoV-II peptide. The twenty epitopes selected for further characterization of immunogenicity and cross-reactivity are presented in (Table 3). In this presentation, peptides are named by their start to stop positions in the SARS-CoV-II spike protein sequence.

**Table 3:** Twenty Selected Epitopes The final twenty peptides from the combination of the DRFU and RF methods to determine candidates for vaccination trial. The identifier of the peptide consists of the start to stop position within the sequence of the spike protein from ancestral SARS-CoV-II. The peptides are sorted by starting position.

DRFU Selected Peptides		RF Selected Peptides	
Position	Sequence	Position	Sequence
0097-0111	KSNIIRGWIFGTTLD	0169-0183	EYVSQPFLMDLEGKQ
0101-0115	IRGWIFGTTLDSKTQ	0493-0507	QSYGFQPTNGVGYQP
0193-0207	VFKNIDGYFKIYSKH	0541-0555	FNFNGLTGTGVLTES
0345-0359	TRFASVYAWNRKRIS	0665-0679	PIGAGICASYQTQTN
0441-0455	LDSKVGGNYNYLYRL	0745-0759	DSTECSNLLLQYGSF
0553-0567	TESNKKFLPFQQFGR	0825-0839	KVTLADAGFIKQYGD
0621-0635	PVAIHADQLTPTWRV	0885-0899	GWTFGAGAALQIPFA
0777-0791	NTQEVFAQVKQIYKT	0905-0919	RFNGIGVTQNVLYEN
		1069-1083	PAQEKNFTTAPAICH
		1149-1163	KEELDKYFKNHTSPD
		1173-1187	NASVVNIQKEIDRLN
		1245-1259	KGCCSCGSCCKFDED

### Epitope immunogenicity and cross-reactivity

Antibodies induced by each candidate vaccine were evaluated for: a) immunogenicity (reactivity to peptides of the same sequence as the epitope), b) epitope recognition within the parent protein (ancestral SARS-CoV-II spike), c) cross-reactivity across variants (reactivity to Delta and Omicron SARS-CoV-II spike), and d) cross-reactivity across viruses (reactivity to SARS-CoV and MERS-CoV spike). This represents a series of increasingly stringent criteria designed to evaluate the ability of each epitope to induce antibody responses that are reactive to a protective antigen across increasingly evolutionarily distance. ELISA results to each of these targets are presented within the supplementary data: peptide (S2 Fig), ancestral SARS-CoV-II spike (S3 Fig), Delta SARS-CoV-II spike (S4 Fig), Omicron SARS-CoV-II spike (S5 Fig), SARS-CoV spike (S6 Fig), and MERS-CoV spike (S7 Fig).

Across the candidate epitopes, four primary functional categories emerge with respect to immunogenicity and cross-reactivity: i) non-immunogenic, ii) non-reactive with parent protein, iii) reactive with conserved epitope sequence in non-parent protein, and iv) cross-reactive with non-conserved epitopes in non-parent protein (Table 4).

**Table 4:** Patterns of Reactivity. Antibodies corresponding to each of the epitopes were investigated through ELISA against peptides of the same sequence (“Pep” column) as well as spike glycoproteins from the indicated sources. For the peptide ELISAs, green indicates a mean IgG titre of over 1:100 for at least two of the three immunized mice; red indicates a mean IgG titre of over 1:100 for at least two of the three immunized mice. Group I, non-immunogenic epitopes; Group II, non-reactive with parent protein; Group III, recognition of sequence conserved epitopes in non-parent protein; and Group IV, recognition non-conserved epitopes. CS = conserved epitope sequence; DS = divergent epitope sequence.

					Delta		Omicron		SARS-CoV		MERS-CoV	
	Epitope	Method	Pep	Parent Protein	CS	DS	CS	DS	CS	DS	CS	DS
**I**	777-791	DRFU										
	905-919	RF										
**II**	193-207	DRFU										
	441-455	DRFU										
	665-679	RF										
	745-759	RF										
	885-899	RF										
	1069-1083	RF										
	1245-1259	RF										
**III**	97-111	DRFU										
	101-115	DRFU										
	169-183	RF										
	1149-1163	RF										
	1173-1187	RF										
**IV**	345-359	DRFU										
	493-507	RF										
	541-555	RF										
	553-567	DRFU										
	621-635	DRFU										
	825-839	RF										

### Immunogenicity

**Fig 2:**
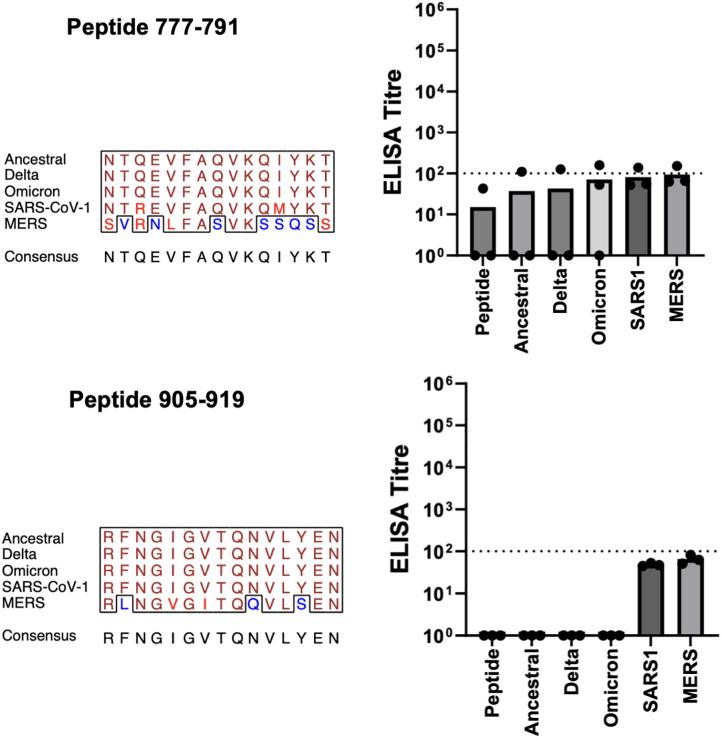
Non-Immunogenic Vaccine Candidates. Balb/c mice (n = 3) of 5−6 weeks of age were immunized three times at four-week intervals. Each immunization consisted of IM delivery of 5 μg of KLH-peptide fusion formulated with 30% Emulsigen-D in a volume of 50 μl (25 μl/thigh). Mice received vaccinations on days 0, 28, and 46. Serum samples obtained on days 0, 28, 42, and 76. ELISAs were performed against peptides of the same sequence as represented in the vaccine or spike protein from the various sources with serum samples collected on Day 76. ELISA titres are shown in the bar graph for the peptide represented in the vaccine, for the complete spike protein from the ancestral, Delta, and Omicron variants of SARS-CoV-II, and finally for the complete spike protein from SARS-CoV-1 and MERS-CoV. Each of three titre values is given by a dot, with the mean being the height of the bar. Multiple sequence alignment of aligned portions of the 5 spike proteins was produced by the EMBOSS prettyplot program (https://bio.tools/prettyplot). The panel title gives the start-to-stop positions of the subject peptide from the spike from ancestral SARS-CoV-II. In the multiple sequence alignment, identical amino acids are shown in brown, conservative substitutions in red, and non-conservative substitutions in blue. Consensus sequence determined by the prettyplot program with default parameters.

Immunogenicity was quantified via ELISAs against peptides of the same sequence as the epitope represented in each candidate vaccine. Most of the vaccines induced high (1:10,000–100,000) epitope-specific IgG antibody titres (S2 Fig). Using a titre of 1:100 as an arbitrary threshold of responsiveness, two of candidate epitopes (777–791 and 905–919) were deemed non-immunogenic (Fig 2). That 90% of the epitopes (18/20) were immunogenic confirms the utility of translation of the peptide epitopes into candidate vaccines through chemical conjugation to a common carrier protein and utilization of an inexpensive adjuvant.

### Reactivity with parent protein

**Fig 3:**
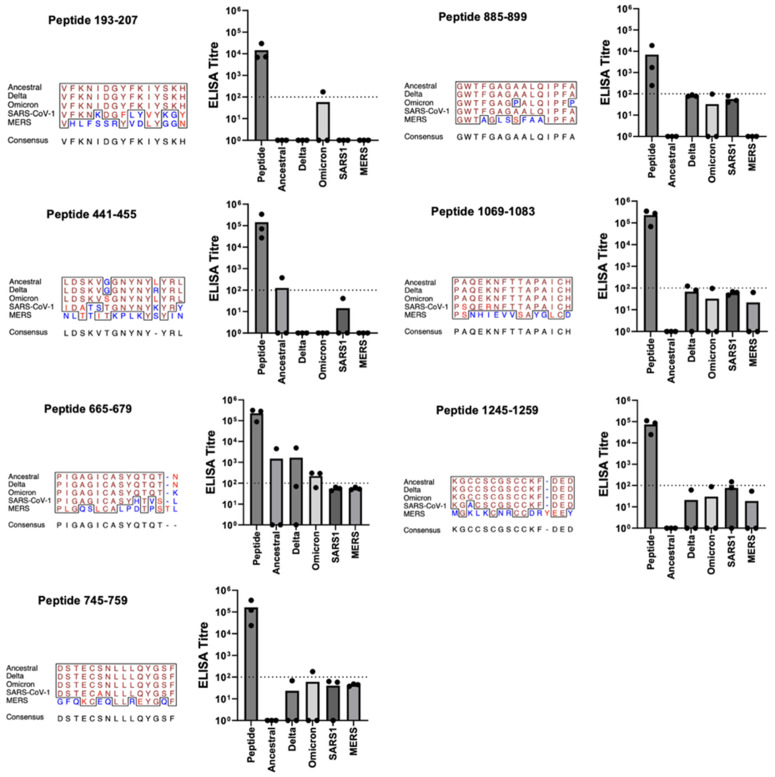
Vaccine Candidates with Non-Reactive Antibodies to Parent Protein. Balb/c mice (n = 3) of 5-6 weeks of age were immunized three times at four-week intervals. Each immunization consisted of IM delivery of 5 μg of KLH-peptide fusion formulated with 30% Emulsigen-D in a volume of 50 μl (25 μl/thigh). Mice received vaccinations on days 0, 28, and 46. Serum samples obtained on days 0, 28, 42, and 76. ELISAs were performed against peptides of the same sequence as represented in the vaccine or spike protein from the various sources with serum samples collected on Day 76. ELISA titres are shown in the bar graph for the peptide represented in the vaccine, for the complete spike protein from the ancestral, Delta, and Omicron variants of SARS-CoV-II, and finally for the complete spike protein from SARS-CoV and MERS-CoV. Each of three titre values is given by a dot, with the mean being the height of the bar. Multiple sequence alignment of aligned portions of the 5 spike proteins was produced by the EMBOSS prettyplot program (https://bio.tools/prettyplot). The panel title gives the start-to-stop positions of the subject peptide from the spike from ancestral SARS-CoV-II. In the multiple sequence alignment, identical amino acids are shown in brown, conservative substitutions in red, and non-conservative substitutions in blue. Consensus sequence determined by the prettyplot program with default parameters.

ELISAs to SARS-CoV-II spike were performed to evaluate recognition of conserved epitope targets in the context of the parent protein. This was to determine, among other factors, the extent to which the linear epitopes are available for binding within the native protein. A more relaxed standard was applied for determining reactivity against proteins than the peptides: a mean IgG titre of over 1:100 for at least two of the three immunized mice. Within the 18 epitope-specific antibodies tested, half were unreactive with the parent protein. This included epitope-specific antibodies against 193–207, 441–445, 541–555, 665–679, 745–759, 885–899, 1069–1083, and 1245–1259 (Fig 3). This presumably reflects shielding of the epitope by structural elements of the protein. Several epitopes whose antibodies lacked reactivity to ancestral spike had degrees of reactivity with spike from SARS-CoV-II variants, as well as the other coronaviruses, likely reflecting subtle differences in protein structures or stabilities which present greater opportunity for antibody binding. These reactivities, however, are low and inconsistent, and unlikely to be of any functional consequence.

### Reactivity with conserved epitopes in non-parent proteins

**Fig 4:**
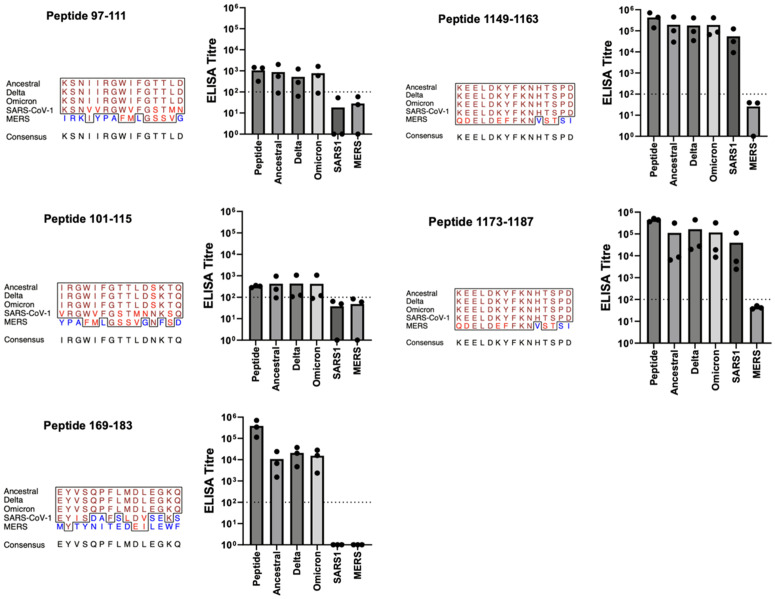
Vaccine Candidates which Induce Antibodies Reactive with the Conserved Epitope. Balb/c mice (n = 3) of 5-6 weeks of age were immunized three times at four-week intervals. Each immunization consisted of IM delivery of 5 μg of KLH-peptide fusion formulated with 30% Emulsigen-D in a volume of 50 μl (25 μl/thigh). Mice received vaccinations on days 0, 28, and 46. Serum samples obtained on days 0, 28, 42, and 76. ELISAs were performed against peptides of the same sequence as represented in the vaccine or spike protein from the various sources with serum samples collected on Day 76. ELISA titres are shown in the bar graph for the peptide represented in the vaccine, for the complete spike protein from the ancestral, Delta, and Omicron variants of SARS-CoV-II, and finally for the complete spike protein from SARS-CoV and MERS-CoV. Each of three titre values is given by a dot, with the mean being the height of the bar. Multiple sequence alignment of aligned portions of the 5 spike proteins was produced by the EMBOSS prettyplot program (https://bio.tools/prettyplot). The panel title gives the start-to-stop positions of the subject peptide from the spike from ancestral SARS-CoV-II. In the multiple sequence alignment, identical amino acids are shown in brown, conservative substitutions in red, and non-conservative substitutions in blue. Consensus sequence determined by the prettyplot program with default parameters.

Cross-reactivity to spike protein from SARS-CoV-II variants and other coronaviruses was evaluated. The first functional category is vaccines that induce antibodies which recognize a conserved epitope sequence in the context of non-parent protein (Table 4). There were five epitope-specific antibodies (97–111; 101–115; 169–183; 1149–1163; 1173–1187) with reactivities to the Delta and Omicron variants (Fig 4). Antibodies to these epitopes were largely unreactive with spike from SARS-CoV and MERS-CoV which have sequence differences in the epitope region.

### Reactivity with non-conserved epitopes

**Fig 5:**
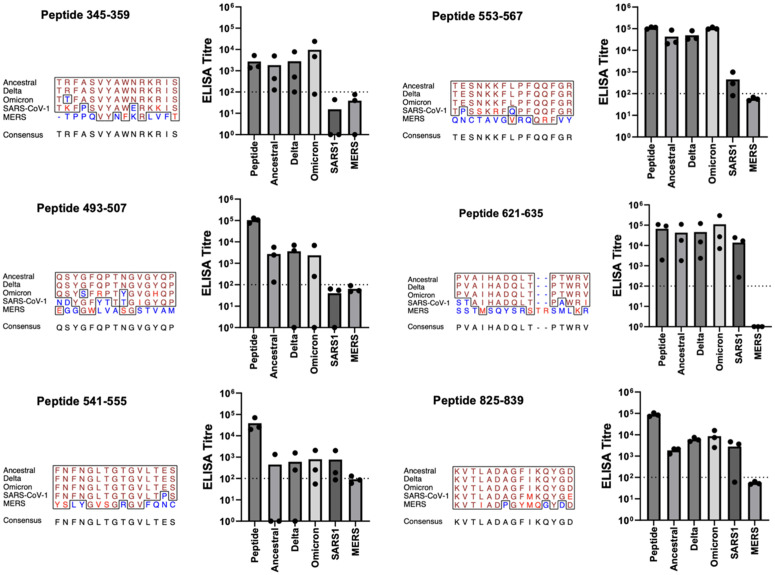
Vaccine Candidates which Antibodies Reactive to Non-Conserved Epitopes. Balb/c mice (n = 3) of 5-6 weeks of age were immunized three times at four-week intervals. Each immunization consisted of IM delivery of 5 μg of KLH-peptide fusion formulated with 30% Emulsigen-D in a volume of 50 μl (25 μl/thigh). Mice received vaccinations on days 0, 28, and 46. Serum samples obtained on days 0, 28, 42, and 76. ELISAs were performed against peptides of the same sequence as represented in the vaccine or spike protein from the various sources with serum samples collected on Day 76. ELISA titres are shown in the bar graph for the peptide represented in the vaccine, for the complete spike protein from the ancestral, Delta, and Omicron variants of SARS-CoV-II, and finally for the complete spike protein from SARS-CoV and MERS-CoV. Each of three titre values is given by a dot, with the mean being the height of the bar. Multiple sequence alignment of aligned portions of the 5 spike proteins was produced by the EMBOSS prettyplot program (https://bio.tools/prettyplot). The panel title gives the start-to-stop positions of the subject peptide from the spike from ancestral SARS-CoV-II. In the multiple sequence alignment, identical amino acids are shown in brown, conservative substitutions in red, and non-conservative substitutions in blue. Consensus sequence determined by the prettyplot program with default parameters.

The highest potential epitopes are those which induce antibodies whose reactivities persist to non-conserved epitopes in non-parent proteins (Table 4). A panel of six epitope-specific antibodies (345–359; 493–507; 541–555; 553–567; 621–635; 825–839) a third of the immunogenic starting group, displayed cross reactivities across a range of spike proteins, including a tolerance for sequence variations within the target epitope (Fig 5).

## Discussion

While the impacts of SARS-CoV-II were extreme, they are by no means isolated. The SARS outbreak of 2003 infected approximately 8,000 people, killed close to 800, and cost the global economy over $40 billion [31]. Recent outbreaks of Ebola, Zika, and MERS-CoV were responsible for thousands of fatalities and considerable economic losses [32,33]. The 2009 H1N1 flu pandemic infected hundreds of millions of people causing hundreds of thousands of deaths [34]. Other influenza strains threaten to jump species barriers with potential for high morbidity and mortality, including recent concerns of transmission of H5N1 to humans [35,36].

Vaccines are the most effective means to control infectious diseases; however, classical approaches to vaccine development typically take 7–10 years. Global organizations, like the World Health Organization (WHO) and the Coalition for Epidemic Preparedness and Innovations (CEPI), aim to reduce this response time to 100 days after the pathogen is identified [37]. While this would significantly improve responsiveness to emerging diseases, it still lags well behind the time frames in which new pathogens can emerge and inflict damage. Traditional approaches to vaccine development are grounded in the induction of highly specific immune responses. This introduces a significant, non-compressible time barrier as specific targets are only revealed after the pathogen emerges. This restricts the capacity to prepare vaccines against unrealized, though inevitable, future threats. Priority on specificity also restricts the opportunity for cross-protection against other related pathogens, emerging variants of the original pathogen, or antigenic variants of an existing pathogen. As such, while highly specific vaccines based on well-described targets are a logical strategy for existing diseases of stable and defined pathogens, this approach may not be ideal for rapid responses to emerging pathogens. Alternatively, through greater understanding of mechanisms of cross-protection it may be possible to develop vaccines that enhance this broader range of protection.

Humoral (antibody) responses are central to the protection afforded by most vaccines. For example, neutralizing antibodies are the critical correlate of protection to pathogenic coronaviruses such as SARS-CoV and MERS-CoV [38–43]. In addition to being key effectors of protection, antibodies, with their vast, complex, and dynamic populations, are also well suited for high throughput investigations. Serum immunoglobulin G (IgG) antibody populations have capacity for recognition of over a quadrillion (10^15) unique targets enabling highly nuanced immunological responses which are ideal for epitope discovery [44]. Further, the capacity of antibody-secreting cells to rapidly generate copious copies of specific antibodies [24] provides a robust, dynamic signal to characterize immunological responses. Lastly, the structure of IgG antibodies, consisting of unique, specificity-determining regions of the Fab arms as well as a structurally conserved Fc region, enables a common detection method, independent of binding specificities.

In the current investigation, a commercial immunoarray representing the proteomes of SARS-CoV-II, SARS-CoV, and MERS-CoV was used to characterize serum antibody populations from ferrets experimentally infected with SARS-CoV-II. The immediate priority was to identify epitopes with enhanced cross-reactivity against spike glycoprotein. A further reaching goal was to establish a pipeline through which immunoarrays can serve as a foundation for the discovery of cross-reactive epitopes. While immunoarrays are an appealing tool to characterize antibody reactivities, the volume and complexity of the associated data complicates the extraction of meaningful biological information. We developed a program called EPIphany (“EPItope arrays Pose Hard ANalYsis problems”) to provide an intuitive user interface to analyze immunoarray data [27].

Here we build on the EPIphany platform to apply two different bioinformatic approaches to select top candidate epitopes for the development of cross-reactive vaccines. From a functional perspective, the DRFU method is more accessible to biologists and can be performed with minimal bioinformatics resources. This type of analysis is also already freely available in established software platforms [27]. The random forest (RF) method presents a barrier to those not familiar with machine learning or R but was chosen for its power and semi-interpretability. Although considered a black-box technique, RF is semi-interpretable in a biological context, as the peptides (features) that are most indicative of whether a sample belongs to the control or treatment group can be determined according to the chosen metrics. By applying a more interpretable technique (compared to neural networks, for example) of machine learning, we were able to obtain from the trained model the features (sequences of potential epitopes) used to make successful predictions. Each method is simple enough that they could be run in parallel and, given that the methods prioritized largely unique epitopes, and that each method identified epitopes with promising cross-reactivity, there may be value in employing both approaches.

With priority to enable rapid, high throughput screening, there is a necessity for a simple procedure for translation of the peptide epitopes into vaccines. Here, chemical conjugation of the epitope peptides to a commonly utilized carrier protein, KLH, was employed. Kits for chemical conjugation of peptide epitopes to KLH are commercially available and require no specialized training or equipment. Of the peptides investigated, 90% induced high (>1:100) titre epitope-specific responses, which verifies the utility of this approach.

A potential challenge to epitope identification through immunoarrays is that the technology is limited to linear epitopes, whereas most neutralizing antibodies are associated with conformational epitopes representing non-sequential residues within the protein antigen. There are, however, examples of protective antibody responses to linear epitopes and recent computational advancements have even pioneered strategies to mimic conformational epitopes with linear peptide sequences [45]. For linear epitopes to afford protection it is essential that the associated antibodies can bind their targets in the context of higher-order protein structure. Within the panel of epitopes investigated, eleven of eighteen immunogenic epitopes induced antibodies that were reactive with the parent protein, an essential prerequisite for neutralizing potential.

The extent of reactivity of the antibody populations induced by the various epitopes was investigated via *in vitro* assays. A schematic overview of the pipeline is presented (Fig 1). The *in vitro* screening steps function as filters to prioritize promising epitopes for eventual testing in animal challenge models. The results of the current study highlight the various ways in which the selected epitopes can fall short of the final objectives.

The first selection filter highlights epitopes that are non-immunogenic, at least under our standardized approaches of formulation and delivery. Further efforts could be employed to improve the immunogenicity of these epitopes, including application of software platforms to enable rational epitope expansion to better represent B cells epitopes [46]. This is especially relevant given that the DRFU and RF methods provided 14 and 15 candidates, respectively, which were reduced to 8 and 12 by identifying sets of overlapping peptides and selecting only one representative from each set. Choosing a different representative, i.e. a peptide neighboring the one that was selected, might have resulted in a stronger immunological response. With this approach, there is the danger that the expansions alter the characteristics that led to their initial selection. Alternate approaches of formulation and delivery, including consideration of various carrier proteins and adjuvants, could also be attempted, although this would significantly increase experimental cost and complexity. More optimistically, that 90% of the epitopes under consideration were translated into immunogenic vaccines through a non-specialized approach provides a simple and effective way for labs with limited experience in vaccine development to rapidly translate candidate epitopes into vaccines.

The second filter is to identify epitopes whose antibodies are unreactive with the parent protein. This likely reflects shielding of the epitope, or portions of the epitope, by the protein structure to limit antibody binding. For the current investigation, immunogenic epitopes whose antibodies failed to react with the ancestral spike protein could be rationalized with consideration of the known structure of the protein. In instances where a detailed structure of the priority antigen is known it would be possible to confirm epitope surface accessibility. For many pathogens, particularly those that are newly emerging and highly-novel, this level of structural information is unlikely to be available.

The third selection criterion is identification of epitope-associated antibodies which bind conserved epitopes within the context of biologically relevant derivatives of the parent antigen. The inability to bind conserved sequences in different protein contexts likely reflects sequence differences outside the core epitope that cause structural changes that impede antibody binding. Within the panel of twenty epitopes, we identified five which demonstrated reactivity with both SARS-CoV-II variants, as well as two which were also reactive with the conserved epitope sequence within spike from SARS-CoV. Notably, none of the 18 immunogenic epitopes had conserved sequences with MERS-CoV.

The fourth and final stage is the identification of epitopes whose antibodies can react with non-conserved epitopes within the context of non-parent proteins. Our investigation revealed five epitopes whose cross-reactivity included spike from SARS-CoV with sequence differences within the core epitope as well as one with reactivity to MERS-CoV spike. The declining number of cross-reactive epitopes with increasing evolutionary distance begins to highlight some of the potential limitations to the extent of cross-reactivity, and cross-protection, that can be achieved.

Within the current investigation cross-reactivity was examined as an academic exercise for the purpose of creating a pipeline. Had the goal been focussed on development of protective vaccines, it would have been beneficial to restrict the epitopes under consideration to the receptor binding domain. Further, with the more ambitious goal of creation of a pan-coronavirus vaccine, a logical next step would be to incorporate virus neutralization assays, which would provide valuable information, in a relatively high-throughput fashion, for prioritizing epitopes for vaccine efficacy trials in animal models of infection.

Finally, we offer several considerations and recommendations for application of this pipeline. The first is to exercise caution in interpreting ELISA antibody titres for prioritizing epitopes, largely because the antibody titres required to achieve protection will be unique to each pathogen and epitope. As information on protective thresholds would be unavailable for an emerging pathogen, it is impossible to set a definitive, meaningful threshold titre; a high IgG titre response to one epitope could be unprotective while a low IgG titre response to another is protective. The extent to which titres can be interpreted can also be complicated by variability of responses to epitopes across individuals. The more reliable approach may be to operate from the assumption that cross-protection depends on cross-reactivity such that antibody populations that are unable to bind the protective antigen of a new pathogen are unlikely to be protective. Further, cross-reactivity does not necessarily ensure cross-protection. From this perspective, ELISA results are best used on a “pass/fail” criteria for elimination of candidate epitopes. Once a list of epitopes is refined from the original candidate pool, it would be valuable to expand the pipeline to include selection criteria based on functional assays of known correlates of protection. This should be approached with an awareness of the potential for a disconnect due to the epitopes occurring within non-neutralizing regions or due to the requirement for conformational epitopes or the requirement for a multivalent approach representing targets with additive or synergistic benefit.

## Supporting information

S1 Fig Representative Array Image: RepliTope™ Antigen Collection Pan-Coronavirus commercial microarrays incubated with serum from experimentally infected SARS-CoV-II convalescent ferrets. Serum was diluted 1:100 in diluent and incubated for 2 h. Each array was washed with 5 exchanges of TBS-T, and once with sterile deionized distilled water. Serum IgG antibodies were detected using Alexa Fluor 647 conjugated goat anti-ferret IgG, Fc(gamma) fragment specific antibody. Peptide arrays were imaged using a GenePix Professional 4200A microarray scanner equipped with a 635 nm laser and fluorescence captured using a 655–695 nm filter. Images were scanned at 10 μm resolution and data acquired using GenePix software (version 7.0). (TIF)

S2 Fig Immunogenicity of Candidate Epitopes: Balb/c mice (n = 3/group) of 5-6 weeks of age were immunized three times at four-week intervals. Each immunization consisted of IM delivery of 5 ug of KLH-peptide fusion formulated with 30% Emulsigen-D in a total volume of 50 ul (25 ul/thigh). Mice received vaccinations on days 0, 28, and 46. Serum samples were obtained on days 0, 28, 42, and 76. ELISAs were performed against the same peptide as represented in the vaccine using serum collected on Day 76. Y-axis is the ELISA titre in log scale. Peptides are shown on the x-axis via their start to stop positions on the spike protein from the ancestral strain of SARS-CoV-II. The readings for each peptide are shown with different symbols. The short horizontal bar indicates the mean of the three titres. (TIF)

S3 Fig Reactivity with Ancestorial Spike: Balb/c mice (n = 3/group) of 5-6 weeks of age were immunized three times at four-week intervals. Each immunization consisted of IM delivery of 5 ug of KLH-peptide fusion formulated with 30% Emulsigen-D in a total volume of 50 ul (25 ul/thigh). Mice received vaccinations on days 0, 28, and 46. Serum samples were obtained on days 0, 28, 42, and 76. ELISAs were performed against the S1S2 domain of Ancestral SARS-CoV-II using serum collected on Day 76. Y-axis is the ELISA titre in log scale. Peptides are shown on the x-axis via their start to stop positions on the spike protein from the ancestral strain of SARS-CoV-II. The readings for each peptide are shown with different symbols. The short horizontal bar indicates the mean of the three titres. (TIF)

S4 Fig Reactivity with SARS-CoV-II Variants: Balb/c mice (n = 3/group) of 5-6 weeks of age were immunized three times at four-week intervals. Each immunization consisted of IM delivery of 5 ug of KLH-peptide fusion formulated with 30% Emulsigen-D in a total volume of 50 ul (25 ul/thigh). Mice received vaccinations on days 0, 28, and 46. Serum samples were obtained on days 0, 28, 42, and 76. ELISAs were performed against Delta Spike using serum collected on Day 76. Y-axis is the ELISA titre in log scale. Peptides are shown on the x-axis via their start to stop positions on the spike protein from the ancestral strain of SARS-CoV-II. The readings for each peptide are shown with different symbols. The short horizontal bar indicates the mean of the three titres. (TIF)

S5 Fig Reactivity with SARS-CoV-II Variants: Balb/c mice (n = 3/group) of 5-6 weeks of age were immunized three times at four-week intervals. Each immunization consisted of IM delivery of 5 ug of KLH-peptide fusion formulated with 30% Emulsigen-D in a total volume of 50 ul (25 ul/thigh). Mice received vaccinations on days 0, 28, and 46. Serum samples were obtained on days 0, 28, 42, and 76. ELISAs were performed against Omicron Spike using serum collected on Day 76. Y-axis is the ELISA titre in log scale. Peptides are shown on the x-axis via their start to stop positions on the spike protein from the ancestral strain of SARS-CoV-II. The readings for each peptide are shown with different symbols. The short horizontal bar indicates the mean of the three titres. (TIF)

S6 Fig Reactivity with SARS-CoV-II Variants: Balb/c mice (n = 3/group) of 5-6 weeks of age were immunized three times at four-week intervals. Each immunization consisted of IM delivery of 5 ug of KLH-peptide fusion formulated with 30% Emulsigen-D in a total volume of 50 ul (25 ul/thigh). Mice received vaccinations on days 0, 28, and 46. Serum samples were obtained on days 0, 28, 42, and 76. ELISAs were performed against SARS-CoV-I Spike using serum collected on Day 76. Y-axis is the ELISA titre in log scale. Peptides are shown on the x-axis via their start to stop positions on the spike protein from the ancestral strain of SARS-CoV-II. The readings for each peptide are shown with different symbols. The short horizontal bar indicates the mean of the three titres. (TIF)

S7 Fig Reactivity with SARS-CoV-II Variants: Balb/c mice (n = 3/group) of 5-6 weeks of age were immunized three times at four-week intervals. Each immunization consisted of IM delivery of 5 ug of KLH-peptide fusion formulated with 30% Emulsigen-D in a total volume of 50 ul (25 ul/thigh). Mice received vaccinations on days 0, 28, and 46. Serum samples were obtained on days 0, 28, 42, and 76. ELISAs were performed against SARS and MERS Spike using serum collected on Day 76. Y-axis is the ELISA titre in log scale. Peptides are shown on the x-axis via their start to stop positions on the spike protein from the ancestral strain of SARS-CoV-II. The readings for each peptide are shown with different symbols. The short horizontal bar indicates the mean of the three titres. (TIF)
