# Supplementary figures and images for: Peptide immunoarrays for rationale development of vaccines with enhanced cross-reactivity
Source: PLoS One. 2025 Sep 4;20(9):e0330741. doi: 10.1371/journal.pone.0330741 (PMC12410791; doi:10.1371/journal.pone.0330741)

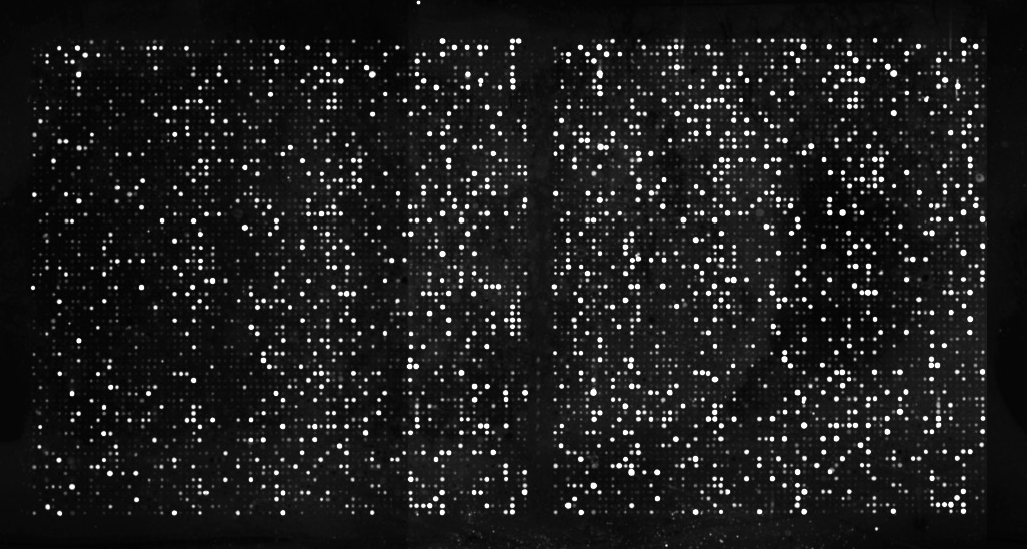

Supplement: S1 Fig — (TIF) [file pone.0330741.s001.tiff]

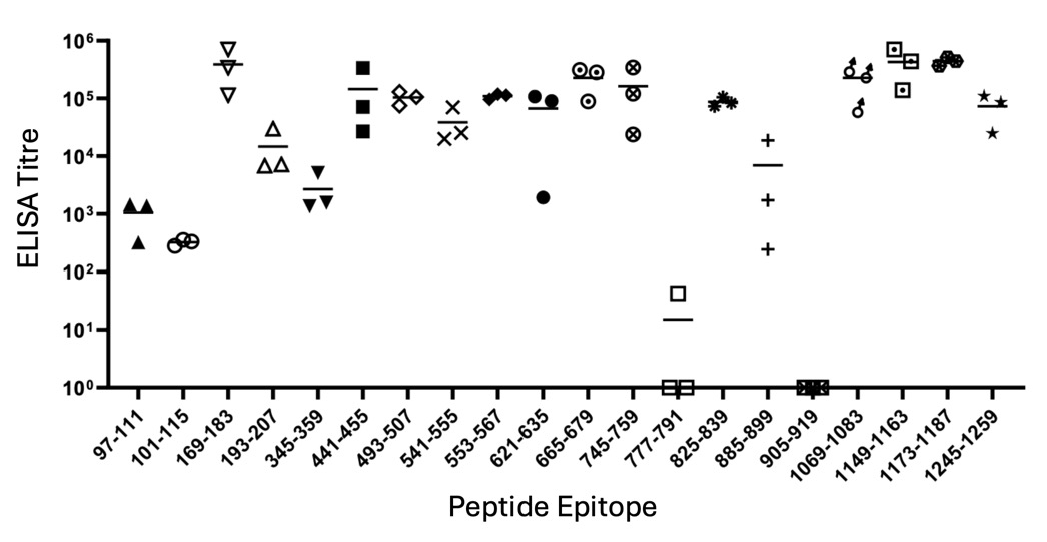

Supplement: S2 Fig — (TIF) [file pone.0330741.s002.tiff]

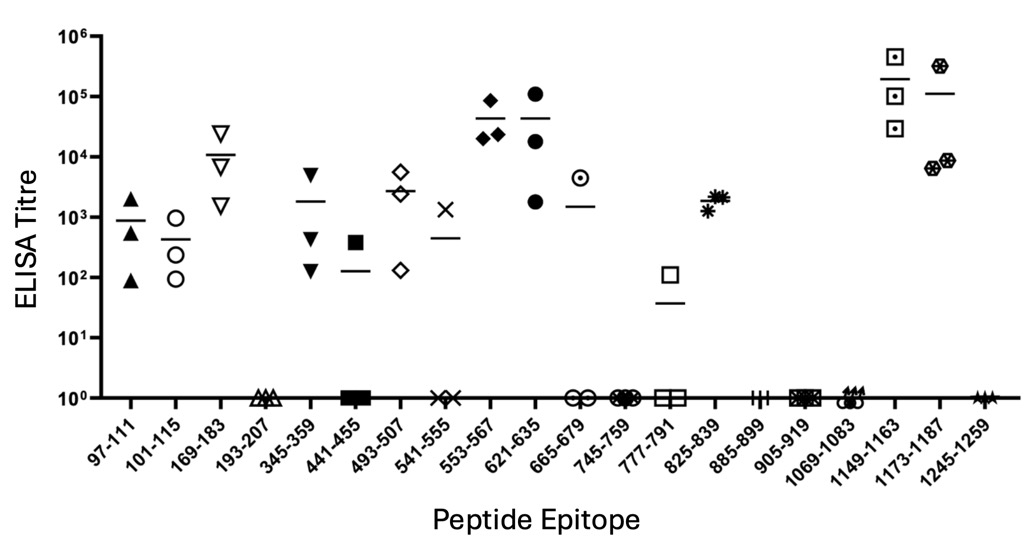

Supplement: S3 Fig — (TIF) [file pone.0330741.s003.tiff]

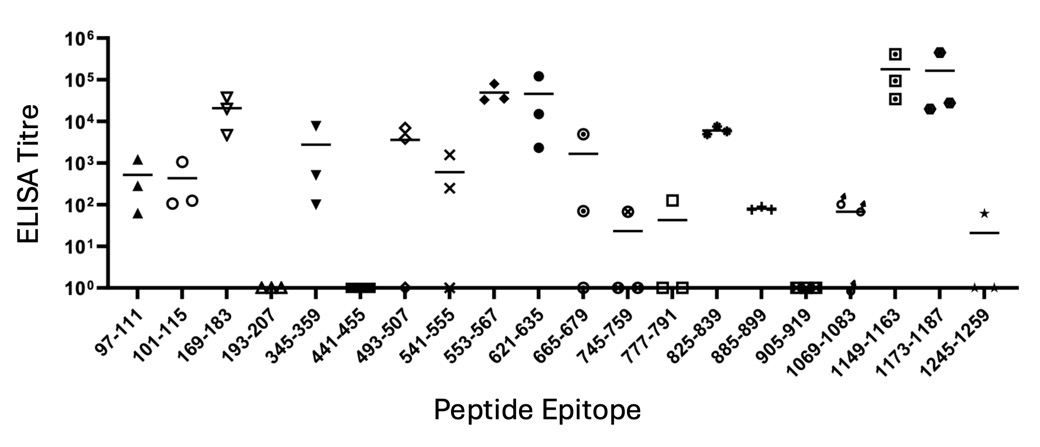

Supplement: S4 Fig — (TIF) [file pone.0330741.s004.tiff]

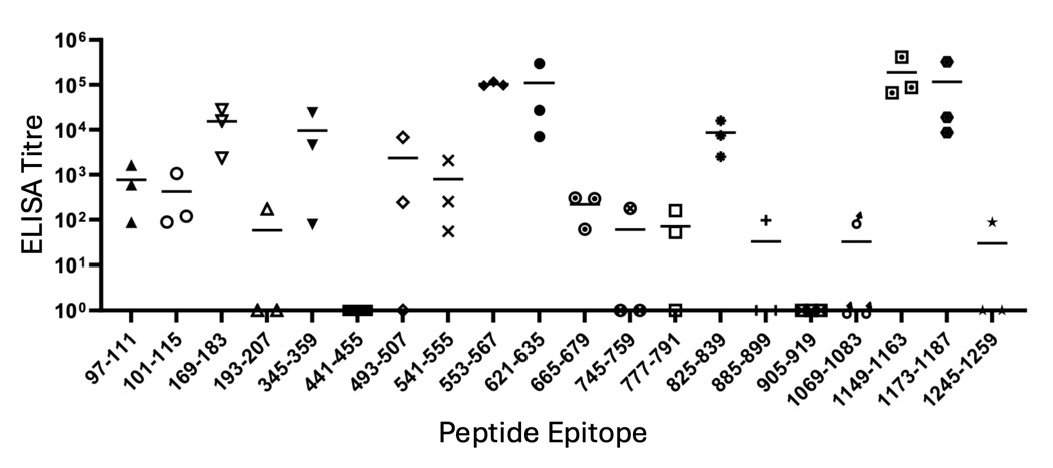

Supplement: S5 Fig — (TIF) [file pone.0330741.s005.tiff]

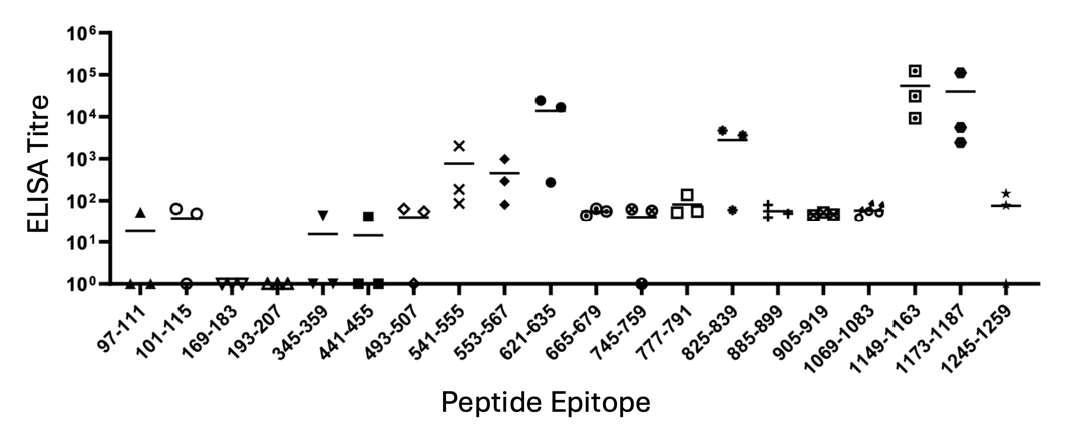

Supplement: S6 Fig — (TIF) [file pone.0330741.s006.tiff]

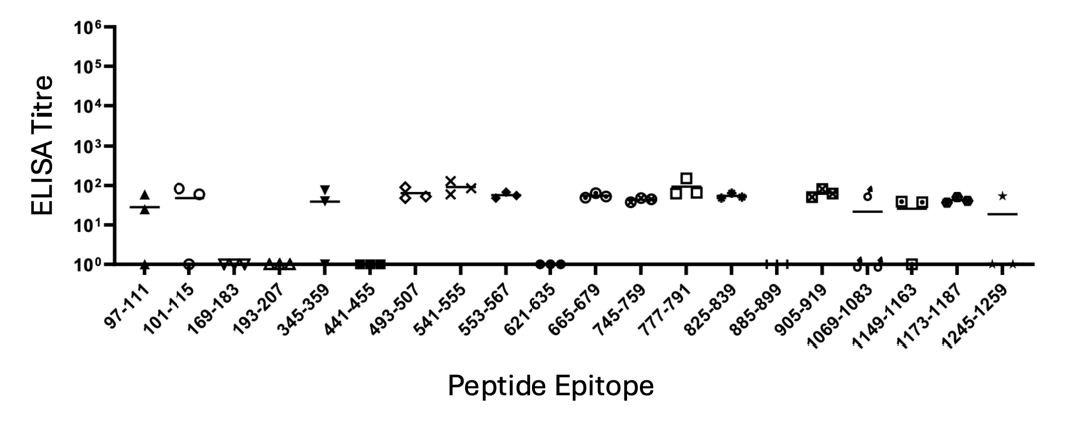

Supplement: S7 Fig — (TIF) [file pone.0330741.s007.tiff]
